# A Cure for Hydrophobia

**Published:** 1874-06

**Authors:** 


### A CURE FOR HYDROPHOBIA.

THE *Salut Public* of Lyons, says Dr. Buisson, claims to have discovered a remedy for this terrible disease ,and to have applied it with complete success in many cases. In attending a female patient in the last stage of canine madness, the doctor imprudently wiped his hands with a handkerchief impregnated with her saliva. There happened to be a slight abrasion on the index finger of the left hand, and, confident in his own curative system, the doctor merely washed the part with water. He was fully aware, however, of the imprudence he had committed, and gives the following account of the matter afterward : " Believing that the malady would not declare itself till the fortieth day, and having numerous patients to visit, I put off from day to day the application of my remedy—that is to say, vapor baths. The ninth day, being in my cabinet, I felt at once a pain in my throat and still greater pain in my eyes. My body felt so light that I felt as if I could jump to a prodigious height, or, if thrown out of a window, I could sustain myself in the air. My hair was so sensitive that I appeared to be able to count each separately without looking at it. Saliva kept continually forming in my mouth. Any movement of air caused great pain to me, and I was obliged to avoid the sight of brilliant objects. I had a continual desire to run and bite—not human beings, but animals, and all that was near me. I drank with difficulty, and I remarked that the sight of water distressed me more than the pain in my throat. I believe that by shutting the eyes any one suffering from hydrophobia can always drink. The fits came on every five minutes, and I then felt the pain start from the index finger and run up the nerves to the shoulder. In this state, thinking that my course was preservative, not curative, I took a vapor bath, not with the intention of cure, but of suffocating myself. When the bath was at the heat of 52 centigrade (93.3.5 Fahrenheit), all the symptoms disappeared as if by magic, and since then I have never felt anything more of them. I have attended more than eighty persons bitten by mad animals ; I have not lost a single one." When a person is bitten by a mad dog, he must, for seven successive days, take a vapor bath—" *a la Russe*," as it is called—of 57 to 63 degrees. This is the preventive remedy. A vapor bath may be quickly made by putting three or four red hot bricks in a bucket or tub of water, and let the patient sit over it on a cane-bottomed or willow chair, enveloped in a large blanket, for fifteen or twenty minutes. When the disease is declared, it only requires one vapor bath, rapidly increasing to 37 centigrade, then slowly to 53; and the patient must strictly confine himself to his chamber until the cure is complete.

